# Ten recommendations for sarcoma surgery: consensus of the surgical societies based on the German S3 guideline “Adult Soft Tissue Sarcomas”

**DOI:** 10.1007/s00423-023-03002-3

**Published:** 2023-07-11

**Authors:** Jens Jakob, Dimosthenis Andreou, Jens Bedke, Dominik Denschlag, Hans Roland Dürr, Steffen Frese, Thomas Gösling, Thomas Graeter, Viktor Grünwald, Robert Grützmann, Jürgen Hoffmann, Ingolf Juhasz-Boess, Bernd Kasper, Vlada Kogosov, Wolfram Trudo Knoefel, Burkhard Lehner, Marcus Lehnhardt, Lars H. Lindner, Cordula Matthies, Jalid Sehouli, Selma Ugurel, Peter Hohenberger

**Affiliations:** 1 Sarcoma Unit, Department of Surgery and Mannheim Cancer Center, University Medical Center Mannheim, University of Heidelberg, Theodor-Kutzer-Ufer 1-3, D-68135 Mannheim, Germany; 2 Department of Orthopedics and Trauma, Medical University of Graz, Graz, Austria; 3 Department of Urology, Klinikum Stuttgart, Stuttgart, Germany; 4 Department of Gynecology, Hochtaunus-Kliniken gGmbH, Bad Homburg, Germany; 5 Orthopaedic Oncology, Department of Orthopaedics and Trauma Surgery, LMU Klinikum, University Hospital, LMU Munich, Munich, Germany; 6 AMEOS Klinikum Schönebeck, Schönebeck, Germany; 7 Department of Trauma and Orthopedic Surgery, Clinical Center Braunschweig, Braunschweig, Germany; 8 Thoracic Surgery, SLK-Lungenklinik Lowenstein, Lowenstein, Germany; 9 Clinic for Urology, Clinic for Medical Oncology, University Hospital Essen, Essen, Germany; 10 Department of Surgery, Comprehensive Cancer Center, University Hospital of Erlangen, Friedrich-Alexander-University Erlangen-Nürnberg (FAU), Erlangen, Bavaria Germany; 11 Department of Oral and Maxillofacial Surgery, University Hospital Heidelberg, Heidelberg, Germany; 12 Department of Gynecology, Obstetrics and Reproductive Medicine, University of Freiburg Faculty of Medicine, Freiburg im Breisgau, Germany; 13 Sarcoma Unit, Mannheim Cancer Center (MCC), Mannheim University Medical Center, University of Heidelberg, Mannheim, Germany; 14 Comprehensive Cancer Center Göttingen G-CCC, University Medical Center Göttingen, Georg August University, Göttingen, Germany; 15 CCC-N (Comprehensive Cancer Center Lower Saxony), Göttingen, Germany; 16 Department of Surgery, Heinrich-Heine-University and University Hospital Duesseldorf, Duesseldorf, Germany; 17 Department of Orthopaedics, Section Orthopedic Oncology and Septic Orthopedic Surgery, Heidelberg University Hospital, Heidelberg, Germany; 18 Department of Plastic Surgery, BG University Hospital Bergmannsheil, Ruhr University Bochum, Bochum, Germany; 19 Department of Medicine III, University Hospital, Ludwig Maximilian University Munich, Munich, Germany; 20 Department of Neurosurgery, University Hospital and Julius-Maximilians-University, Wuerzburg, Germany; 21 Department of Gynecology with Center for Oncological Surgery, Charité Comprehensive Cancer Center, University Medicine Berlin, Berlin, Germany; 22 Department of Dermatology, University Hospital Essen, University of Duisburg-Essen, Essen, Germany; 23 Division of Surgical Oncology and Thoracic Surgery, Department of Surgery, University Hospital Mannheim, University of Heidelberg, Mannheim, Germany

**Keywords:** Sarcoma guideline, Evidence, Surgical recommendation, Delphi process

## Abstract

**Purpose:**

The evidence-based (S3) guideline “Adult Soft Tissue Sarcomas” (AWMF Registry No. 032/044OL) published by the German Guideline Program in Oncology (GGPO) covers all aspects of sarcoma treatment with 229 recommendations. Representatives of all medical specialties involved in sarcoma treatment contributed to the guideline. This paper compiles the most important recommendations for surgeons selected by delegates from the surgical societies.

**Methods:**

A Delphi process was used. Delegates from the surgical societies involved in guideline process selected the 15 recommendations that were most important to them. Votes for similar recommendations were tallied. From the resulting ranked list, the 10 most frequently voted recommendations were selected and confirmed by consensus in the next step.

**Results:**

The statement “Resection of primary soft tissue sarcomas of the extremities should be performed as a wide resection. The goal is an R0 resection” was selected as the most important term. The next highest ranked recommendations were the need for a preoperative biopsy, performing preoperative MRI imaging with contrast, and discussing all cases before surgery in a multidisciplinary sarcoma committee.

**Conclusion:**

The evidence-based guideline “Adult Soft Tissue Sarcomas” is a milestone to improve the care of sarcoma patients in Germany. The selection of the top ten recommendations by surgeons for surgeons has the potential to improve the dissemination and acceptance of the guideline and thus improve the overall outcome of sarcoma patients.

**Supplementary Information:**

The online version contains supplementary material available at 10.1007/s00423-023-03002-3.

## Introduction

Soft tissue sarcomas are a group of rare, heterogeneous tumors that can occur in any body region [1, 2]. Compared with common cancers, the prognosis of rare tumors is worse but has improved overall in recent decades [3]. Centralization of sarcoma treatment represents an opportunity to improve treatment outcomes [4], and cancer registry data show improved local tumor control and local-recurrence-free survival [5–7]. Accreditation of cancer care providers is another strategy to improve outcome. In Germany, in colorectal and pancreatic cancer, administrative data demonstrate improved survival and cost savings at certified cancer centers compared to non-accredited providers [8, 9]. Data from the French NETSARC network confirm these results for sarcoma patients [10–12].

Disease-specific expertise and adherence to the standard of care plays a major role for improved survival in almost all solid cancers. In sarcoma, surgery at expert centers has a positive impact on overall survival of sarcoma patients [11]. Furthermore, data of the European network of excellence CONTICANET and of NETSARC demonstrated that presenting a sarcoma case at the multidisciplinary tumor board (MDT) and adherence to the standard of care have a positive impact on disease-free survival [12, 13]. Consequently, the establishment of certified sarcoma centers and the development of the evidence-based S3 guideline “Adult Soft Tissue Sarcomas” are milestones for the improvement of sarcoma care in Germany. The S3 guideline is a powerful tool to improve the quality and standardization of procedures for adult soft tissue sarcoma. An optimized distribution of the recommendations of the guideline will improve treatment outcomes in Germany.

While radiation therapy and chemotherapy of sarcoma patients are administered by specialists of the respective disciplines, surgical treatment is the responsibility of different specialties depending on the location of the sarcoma (e.g., orthopedic surgery for extremities, head and neck surgery, gynecological surgery for uterine sarcoma). Consequently, different standards and strategies might be applied for sarcoma care derived often from epithelial cancer at a similar site, i.e., removal of lymph nodes. Furthermore, the experience of surgeons outside of specialized centers is low. This may be one reason why deviations from the standard of care mainly occur during the first diagnostic and treatment steps for sarcoma [13]. Particularly the problem of unplanned surgery without prior imaging and biopsy is well known and described with the term “whoops surgery” [14–16].

For this paper, the delegates of the surgical disciplines and scientific surgical societies defined the most important recommendations for surgeons in a Delphi process. They all had collaborated in working group “Therapy of Localized Sarcomas” during the Guideline development before. In accordance with “Europe’s Beating Cancer Plan” (https://health.ec.europa.eu/system/files/2022-02/eu_cancer-plan_en_0.pdf) and the German “National Cancer Plan” (https://www.bundesgesundheitsministerium.de/fileadmin/Dateien/5_Publikationen/Praevention/Broschueren/Broschuere_Nationaler_Krebsplan.pdf), we want to improve the dissemination and use of cancer guidelines. Therefore, we aim at distributing the selected recommendations and by this way strengthen multidisciplinary sarcoma treatment.

## Methods

### Methodology of the S3 guideline “Adult Soft Tissue Sarcomas”

The S3 guideline “Adult Soft Tissue Sarcomas” was developed within the framework of the German Guideline Program in Oncology (GGPO) of the German Cancer Society (German Cancer Society, German Cancer Aid, AWMF): Soft Tissue Sarcoma Long version 1.1, 2022, AWMF Registration Number: 032/044OL, https://www.leitlinienprogramm-onkologie.de/leitlinien/adulte-weichgewebesarkome/). The methodology of the guideline is based on the rules of the Association of the Scientific Medical Societies (AWMF, https://www.awmf.org/regelwerk/). Leading societies were the German Interdisciplinary Sarcoma Group (GISG, http://www.gisg.de/) and the Arbeitsgemeinschaft Internistische Onkologie (AIO, https://www.aio-portal.de/) of the Deutsche Krebsgesellschaft (DKG). The guideline was funded by the German Cancer Aid as part of the GGPO (https://www.leitlinienprogramm-onkologie.de/german-guideline-program-in-oncology/). PH, BK, and VG coordinated the guideline and 41 medical societies were member to the guideline commission.

Within the guideline committee, 11 working groups (e.g., Working Group 5: Therapy of Localized Soft Tissue Sarcoma) and their delegates decided on clinical questions for a systematic literature search: e.g., influence of resection margin on local recurrence-free and overall survival, influence of resection strategy on local recurrence-free and survival, influence of metastasectomy on progression-free survival. The Institute for Research in Operative Medicine (IFOM, University of Witten-Herdecke, Germany) provided the evidence report to answer the clinical questions which afterwards were converted into recommendations by the working groups (for details see https://www.leitlinienprogramm-onkologie.de/fileadmin/user_upload/Downloads/Leitlinien/Adulte_Weichgewebesarkome/Evidenzbericht_LL_Adulte_Weichgewebesarkome.pdf).

All members of the guideline committee voted on all 229 recommendations individually during two consensus conferences. In each case, the evidence grading, recommendation grading (e.g., strong recommendation), and consensus strength (e.g., strong consensus > 95% of those voting) were indicated by two representatives from AWMF and another two from DKG. Beyond the formal recommendation, background text delivers the pros and cons of the recommendations and refers to current literature.

The English version of the guideline can be approached at https://www.leitlinienprogramm-onkologie.de/german-guideline-program-in-oncology/

### Application of the Delphi process that lead to the ten recommendations for surgery

Delegates of 16 surgical societies who actively participated in the S3 guideline committee were invited to take part in the Delphi process (Working Group Dermatologic Oncology of the German Cancer Society and DDG (ADO), Working Group Gynecological Oncology in the German Cancer Society (AGO), Working Group Oncological Thoracic Surgery of the German Cancer Society (AOT), Working Group Urological Oncology of the German Cancer Society (AUO), Professional Association of German Surgeons (BDC), Professional Association for Orthopedics and Trauma Surgery (BVOU), German Society of Plastic, Reconstructive and Aesthetic Surgeons (DGPRÄC), German Society for General and Visceral Surgery (DGAV), German Society for Gynecology and Obstetrics (DGGG), German Society for Oral and Maxillofacial Surgery (DGMKG), German Society for Neurosurgery (DGNC), German Society for Orthopedics and Orthopedic Surgery (DGOOC), German Society for Thoracic Surgery (DGT), German Society for Trauma Surgery (DGU), Interdisciplinary Working Group on Soft Tissue Sarcomas of the German Cancer Society (IAWS), North-East German Society for Gynecological Oncology (NOGGO)). In addition, members of the working group 5 “Therapy of localized soft tissue sarcoma” and six “treatment of local recurrence” from the Guidelines Committee were invited to take part in the Delphi process.

**Fig. 1:**
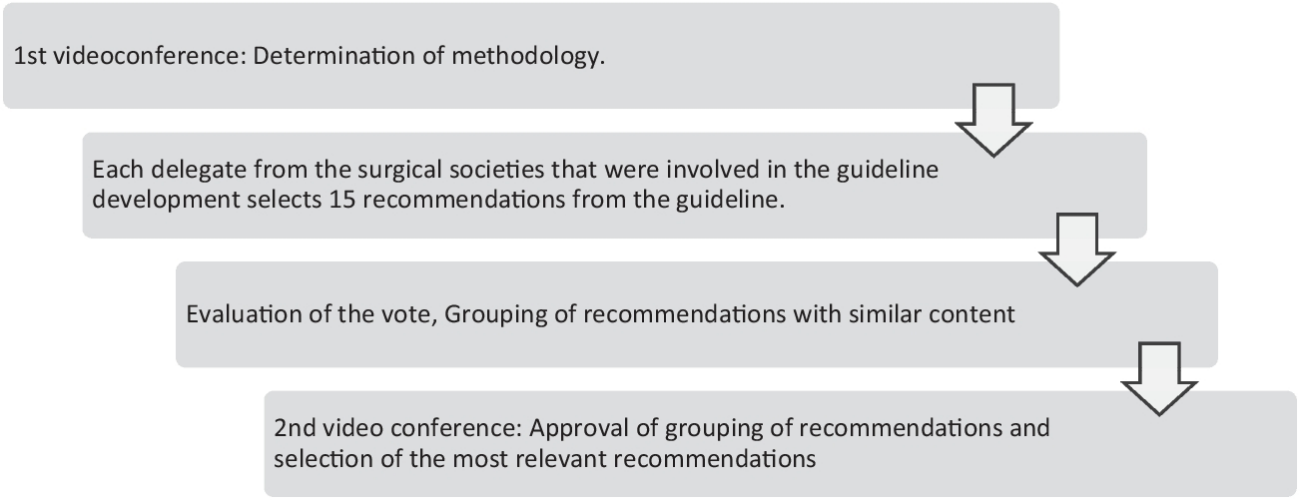
Description of the Delphi method applied

Two joint videoconferences were held. In the first, the methodology of the present work was consented. Hereafter, each delegate selected his/her 15 recommendations out of the 229 considered most important for surgeons (Fig. 1). VK and JJ evaluated the voting and editorially summarized recommendations that were similar in content, adding the individual votes (supplementary table S1). The resulting list was consented in a second videoconference.

## Results

The 17 delegates selected eighty-four different recommendations from the 229 statements in the guideline (supplementary table S2). By combining recommendations with similar content, the number was reduced to 58 statements. The votes for each of those single recommendations were added, and an example how grouping was performed can be found in supplementary table S1.

All selected statements refer to chapter #4 (“Diagnostics, Prognostic Markers and Scores”) and chapter #5 (“Therapy of Localized Soft Tissue Tumors”) of the S3 guideline. In detail, the contents of the recommendations address preoperative biopsy, preoperative imaging, preoperative decision-making, resection strategy, indication for lymph node dissection, handling of the resection specimen, and referral of patients to multidisciplinary sarcoma tumor boards. A detailed summary of the voting results is listed in supplementary table S2.

We rephrased the ten most frequently mentioned recommendations into “action statements for sarcoma surgery” (Table 1).

**Table 1:** Top ten rules for sarcoma surgery. The recommendations were rephrased into instructions and arranged according to their order within a multimodality treatment journey of a virtual sarcoma patient. Referring recommendations of the S3 guideline are given in brackets and are listed in full wording in supplementary table S2

Ten recommendations for sarcoma surgery	
▪ Perform primary excisions only for superficial lumps up to 3cm in size (4.12)	
▪ Arrange for MRI imaging prior to sarcoma resection (4.3)	
▪ Take a biopsy prior to sarcoma resection (4.7)	
▪ Present all sarcoma cases preoperatively to a multidisciplinary tumor board that includes at least one sarcoma expert (5.2)	
▪ Offer preoperative treatment to all stage III sarcoma patients (5.16)	
▪ Perform sarcoma resections as wide resections with the goal of an R0 margin (5.6)	
▪ Strive for an extremity-preserving approach in extremity sarcoma surgery (5.5)	
▪ Do not perform systematic lymphadenectomy without evidence of lymph node metastasis (5.20)	
▪ Make a suture mark on the sarcoma specimen to allow pathologists to obtain a three-dimensional orientation (4.15)	
▪ Refer sarcoma patients to sarcoma centers in the case of R1 resections (5.15)	

## Discussion

The development of the German evidence-based S3 guideline adult soft tissue sarcoma is a milestone for the diagnosis and therapy of sarcoma patients in Germany. To our knowledge, this is the first sarcoma guideline with a systematic literature search and evidence assessment performed by a scientific research institute independent from the disease peers. Over the past decades, standard of care recommendations by ESMO, NCCN, or national scientific groups were developed via task forces or consensus conferences with sometimes more than 50 experts discussing in a multiday, person-to-person meeting [17–20]. Recently, virtual formats had to be adapted due to COVID restrictions [21, 22]. Despite all efforts to integrate scientific data preferably from well-accepted publications during the discussion of a new or to-be-refurbished guideline, these formats open space for personal attitudes or country-specific views.

Forty-one medical societies were involved in the guideline development, sixteen of them representing surgical disciplines mirroring the importance of surgery during sarcoma treatment. Surgeons belong to the main addressees of the guideline since they are frequently the first to see and diagnose a sarcoma patient. Most of the recommendations mentioned here seem self-evident. Data from epidemiological studies, however, indicate that in sarcoma care most deviations from GCP occur in the initial diagnosis and treatment [13, 14]. This effect is likely to be independent of anatomical site. Due to the rarity of sarcomas, surgeons outside of specialized centers — regardless of which discipline they belong to — usually do not have a broad experience in sarcoma care. Furthermore, we cannot expect that the same colleagues study a guideline with over 200 pages and 229 recommendations in detail. Our project intended to improve the knowledge of the basic principles sarcoma care within the surgical community.

The chosen methodology corresponds to a multi-stage structured Delphi survey with the aim of building consensus. Two typical points of criticism of Delphi surveys concern the formulation of the basic theses and the selection of the experts. Here, the basic theses were existing recommendations that merely needed to be prioritized. All experts had been involved in the guideline and were familiar with the subject. Another point of criticism of the methodology could be the grouping process of the recommendations. We grouped these recommendations to avoid subject-specific recommendations from different surgical disciplines, similar in content. All participating experts consented to the method and results of the grouping process. The rephrasing into instructions (Table 1) was done for didactic reasons.

The authors do not aim at elaborating site-specific or specialty-specific differences in sarcoma surgery; thus, only selective comments can be made. The indication and technique of biopsy differs in the recommendations for extremity sarcomas, retroperitoneal sarcomas, and gastrointestinal stromal tumors (Cf. statements 4.7, 5.32, and 10.1 of the guideline). However, there was no doubt that a biopsy is generally preferred to simple tumor excision without biopsy. The advantages of biopsy are obvious. Knowledge of the histology allows shared decision-making and adequate treatment planning. Incisional biopsies should be performed at expert centers to avoid incorrect incisions. Core needle biopsies using coaxial needles are an equi-effective alternative with a lower rate of wound complications [23–25].

**Fig. 2:**
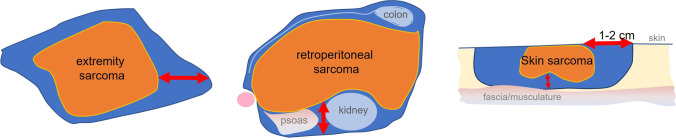
Graphic representation of resection strategies for extremity sarcomas (wide resection), retroperitoneal sarcomas (multivisceral resection), and sarcomas of the skin and subcutaneous tissue. The so-called wide resection requires that the tumor remains covered on all sides by a layer of healthy tissue. The “multivisceral resection” describes how this layer may be achieved under difficult conditions (e.g., by resection the mesocolon and colon, kidney, and psoas together with the tumor). For skin sarcomas, subtype-specific safety margins were defined for the skin surface whereas towards the fascia a micrographic margin control was recommended — which may be achieved by resecting a layer of healthy fascia and musculature

All experts consented that fragmented or unplanned R2 resection should be avoided. This includes the omission of morcellement for uterine soft tissue tumors irrespective of their histology. Regarding surgical strategies, the guideline recommendations reflect specialty-specific aspects as well as surgical traditions. Common to all is the goal of an R0 resection. The strategies used to achieve clear margins are different (Table 1, Fig. 2). For extremity sarcomas, the guideline clearly defines the so-called wide resection (“The tumor remains covered on all sides by a layer of healthy tissue.”). Yet, the resection margin is not specified in centimeters due to the presence of conflicting data [26, 27]. Dermato-surgical recommendations require subtype-specific resection margins due to the different biological behavior of sarcoma subtypes (# 5.30) [26–28]. Margin width for skin or subcutaneous sarcomas are oriented in centimeters to the skin surface but not to be underlying fascia with micrographic margin control. In retroperitoneal sarcoma, deliberate resection of adjacent organs may be necessary to achieve a complete removal of the tumor even if there is no direct evidence of organ infiltration in order to ensure a “layer of healthy tissue around the tumor” (# 5.34) [29]. Thus, the different approaches can be well summarized under the term of a “wide resection with the goal of R0” (Fig. 2). It is a future task of the surgical community to develop more detailed concepts for sarcoma surgery. Subtype-specific resection margins need to be defined with better data prospectively collected.

Due to the low incidence of lymph node metastases in sarcoma, there is no indication for systematic lymphadenectomy without evidence of lymph node metastasis (#5.23) [30]. Adaptation of surgical approaches in head and neck areas as well for gynecological tumors should profit from dissemination of the ten recommendations. Typically, lymph node dissection is often part of gynecological and H&N surgery particularly if no preoperative biopsy has been performed. Epithelial cancers are much more frequent than sarcomas in these disciplines, and removal of the lymph nodes is often part of the standard surgical procedure. Sentinel lymph node biopsy is also not recommended in the German S3 guideline (# 5.31). Other relevant recommendations deal with the performance of organ resections and amputations, the indication for multimodality therapy in stage III, and the referral of patients to certified sarcoma centers (# 5.2, 5.16, 5.5, 5.15).

The ten recommendations point to an important paradigm of the guideline: surgery is an important but not necessarily the most important neither the very first modality in sarcoma treatment. Surgeons of any discipline are very well trained and responsible specialists, however, mainly for other diseases and tumors than sarcomas. In accordance with the National Cancer Plane, it is our mission and responsibility to distribute the knowledge of basic rules in sarcoma treatment in the surgical community.

### Supplementary information

ESM 1 (DOCX 36 kb)

## Data Availability

All data are included in this manuscript and the supplementary material.
