# Supplementary material for: Ten recommendations for sarcoma surgery: consensus of the surgical societies based on the German S3 guideline “Adult Soft Tissue Sarcomas”
Source: Langenbecks Arch Surg. 2023 Jul 11;408(1):272. doi: 10.1007/s00423-023-03002-3 (PMC10333354; doi:10.1007/s00423-023-03002-3)
Supplement: Supplementary file 1 — (DOCX 36 kb) [file 423_2023_3002_MOESM1_ESM.docx]

# Ten Recommendations for Sarcoma Surgery: Consensus of the Surgical Societies based on the German S3 guideline "Adult Soft Tissue Sarcomas".

## Authors

Jens Jakob, Sarcoma Unit, Department of Surgery and Mannheim Cancer Center, University Medical Center Mannheim, University of Heidelberg, Mannheim, Germany, Jens.jakob@umm.de

Dimosthenis Andreou, Department of Orthopedics and Trauma, Medical University of Graz, Graz, Austria, dimosthenis.andreou@medunigraz.at

Jens Bedke, Department of Urology, Eberhard Karls University Tübingen, Tübingen, Germany, Jens.Bedke@med.uni-tuebingen.de

Dominik Denschlag, Department of Gynecology, Hochtaunus-Kliniken gGmbH, Bad Homburg, Germany, Dominik.Denschlag@hochtaunus-kliniken.de

Hans Roland Dürr, Orthopaedic Oncology, Department of Orthopaedics and Trauma Surgery, LMU Klinikum, University Hospital, LMU Munich, Munich, Germany, Hans_Roland.Duerr@med.uni-muenchen.de

Steffen Frese, Department of Thoracic Surgery, Lungenklinik Lostau gGmbH, Lostau, Germany, steffen.frese@email.de

Thomas Gösling, Department of Trauma and Orthopedic Surgery, Clinical Center Braunschweig, Germany, t.goesling@skbs.de

Thomas Graeter, Thoracic Surgery, SLK-Lungenklinik Lowenstein, Lowenstein, Germany, Thomas.Graeter@slk-kliniken.de

Viktor Grünwald, Clinic for Urology, Clinic for Medical Oncology, University Hospital Essen, Essen, Germany, Viktor.Gruenwald@uk-essen.de

Robert Grützmann, Department of Surgery, Comprehensive Cancer Center, University Hospital of Erlangen, Friedrich-Alexander-University Erlangen-Nürnberg (FAU), Erlangen, Germany, Robert.Gruetzmann@uk-erlangen.de

Jürgen Hoffmann, Department of Oral and Maxillofacial Surgery, University Hospital Heidelberg, Heidelberg, Germany, Juergen.Hoffmann@med.uni-heidelberg.de

Ingolf Juhasz-Boess, Department of Gynecology, Obstetrics and Reproductive Medicine, University of Freiburg Faculty of Medicine, Freiburg im Breisgau, Germany, ingolf.juhasz-boess@uniklinik-freiburg.de

Bernd Kasper, Sarcoma Unit, Mannheim Cancer Center (MCC), Mannheim University Medical Center, University of Heidelberg, Mannheim, Germany, Bernd.Kasper@umm.de

Vlada Kogosov, Comprehensive Cancer Center Göttingen G-CCC, University Medical Center Göttingen, Georg August University, Göttingen, Germany; CCC-N (Comprehensive Cancer Center Lower Saxony), vlada.kogosov@med.uni-goettingen.de

Wolfram Trudo Knoefel, Department of Surgery, Heinrich-Heine-University and University Hospital Duesseldorf, Duesseldorf, Germany, Knoefel@med.uni-duesseldorf.de

Burkhard Lehner, Department of Orthopaedics, Heidelberg University Hospital, Heidelberg, Germany, Burkhard.Lehner@med.uni-heidelberg.de

Marcus Lehnhardt, Department of Plastic Surgery, BG University Hospital Bergmannsheil, Ruhr University Bochum, Bochum, Germany, marcus.lehnhardt@bergmannsheil.de

Lars Lindner, Department of Medicine III, University Hospital, Ludwig Maximilian University Munich, Munich, Germany, Lars.Lindner@med.uni-muenchen.de

Cordula Matthies, Department of Neurosurgery, University Hospital and Julius-Maximilians-University, Wuerzburg, Germany, matthies_c@ukw.de

Jalid Sehouli, Department of Gynecology with Center for Oncological Surgery, Charité Comprehensive Cancer Center, University Medicine, Berlin, Germany, jalid.sehouli@charite.de

Selma Ugurel, Department of Dermatology, University Hospital Essen, University of Duisburg-Essen, Germany, Selma.Ugurel@uk-essen.de

Peter Hohenberger, Department of Surgery, Division of Surgical Oncology and Thoracic Surgery, University Hospital Mannheim, University of Heidelberg, Mannheim, Germany, Peter.Hohenberger@umm.de

## Corresponding Author

Professor Jens Jakob, MD

Sarcoma Unit, Department of Surgery and Mannheim Cancer Center

University Medical Center Mannheim, University of Heidelberg

Theodor-Kutzer-Ufer 1-3

D-68135 Mannheim, Germany

Mail jens.jakob@umm.de

phone +49 621 383 3434

Fax +49 621 383 1479

ORCID: 0000-0002-8499-7172

**Supplementary Materials - Index**

| **Supplementary Figures and Tables** |  |
| --- | --- |
| Supplementary table S1 | *pag. 3* |
| Supplementary table S2 | *Pag. 4* |

**Supplementary Figures and Tables**

Supplementary table S1:

Example of the grouping of several recommendations with similar content. The recommend-dation listed as the first is the "grouped recommendation" (items like # 5.6 refer to the numbering of single recommendations in the S3 guideline), compare supplemental table 2).

| These statements were grouped together under the statement 5.6. | 5.6 Resection of primary soft tissue sarcoma of the extremities should be performed as a wide resection. The goal is an R0 resection. |
| --- | --- |
|  | 5.29. Surgical resection in healthy (R0) tissue should be performed for primary cutaneous soft tissue sarcomas without the presence of metastasis. |
|  | 5.34. Complete surgical resection (R0) should be performed as standard therapy for retroperitoneal soft tissue sarcomas. Even without evidence of histologic infiltration of adjacent organs, (portions of) adjacent organs should be removed as part of an en bloc resection. |
|  | 5.41. Complete surgical resection (R0) should be performed as the standard treatment of localized soft tissue sarcomas (not GIST) of the digestive tract. |
|  | 5.51. En bloc resection of the sarcoma (primary tumor or recurrence) in healthy tissue is the basis of any successful reconstructive therapy. |

Supplementary Table 2: Result of the voting on the most important recommendations for surgeons. The statements are given here in the order in which they are mentioned in the guideline. Only recommendations receiving more than one vote are listed.

| **Statement** | **Text of the statement** | **level of evidence (GRADE)** | **Number of votes** | **Summarized with the following recommendations** |
| --- | --- | --- | --- | --- |
| 4.1. | The diagnosis and therapy of soft tissue sarcoma should be performed by or in coordination with a certified sarcoma center or associated cooperation partner. |  | 4 | 5.4 |
| 4.3. | When a malignant soft tissue tumor is suspected, contrast-enhanced magnetic resonance imaging is the primary imaging modality of choice. |  | 5 |  |
| 4.5. | Staging should be performed prior to therapy for soft tissue sarcoma. |  | 4 | 5.32 |
| 4.7. | For histological confirmation of a soft tissue sarcoma, a core needle biopsy or an incisional biopsy should be performed. The decisive factor for the choice of procedure is experience in performing it and in processing and examining the tissue obtained. |  | 12 | 5.1, 5.33, 5.40 |
| 4.8. | If an image-guided core needle biopsy is performed to confirm the diagnosis of a soft tissue tumor, it should be performed using the coaxial technique with ≥16G punches. Multiple cores cylinders should be obtained. |  | 2 |  |
| 4.9. | The biopsy route should be selected so that it can be removed with the definitive resection without widening the resection access. In the case of an incisional biopsy, the biopsy route should always be removed en bloc with the specimen. |  | 3 |  |
| 4.10. | A biopsy should be taken at a specialized center, by or in consultation with the subsequent surgeon. |  | 3 |  |
| 4.12. | Excisional biopsy may be considered for superficial tumors smaller than 3 cm, provided that safe removal of the tumor in healthy tissue is assured. |  | 6 | 5.3 |
| 4.13. | Frozen section examination should not be used for assessment malignancy and subtyping of soft tissue tumors. |  | 4 |  |
| 4.15. | As a minimum requirement, the surgeon should mark the surgically removed resection specimen with thread markings in such a way that three-dimensional orientation is possible for the pathologist. If necessary, a schematic drawing of the orientation should be included. |  | 7 | 5.60, 5.64 |
| 4.16. | In the case of macroscopically close resection, if detected or suspected intraoperatively, the margins of deposition should be marked on the resection specimen. |  | 3 | 5.14, 5.73 |
| 4.18. | If logistically possible, the tumor on the resection specimen should be transferred directly to the pathology department in its native, unfixed state to enable immediate and optimal further processing. Otherwise, the specimen should be transferred to pathology in a sufficient amount of 4% buffered formalin as quickly as possible. |  | 3 |  |
| 4.21. | The minimum distances to relevant resection margins and critical structures should be specified in the histopathological findings. R0 status is defined as „no ink on tumor“. |  | 2 |  |
| 4.27. | The grading of sarcomas should be entity-dependent using the European preferred FNCLCC system (Fédération Nationale des Centres de Lutte Contre le Cancer) or according to the NCI system (National Cancer Institute). The three parameters relevant for this, degree of differentiation, mitotic count per 10 HPFs and extent of necrosis, should be taken from the findings. |  | 2 |  |
| 5.2. | The planning of the treatment of sarcomas should take place in the interdisciplinary tumor board before treatment is instigated. At least one surgical discipline with a focus on soft tissue sarcomas, as well as hematology/oncology, pathology, radiology and radiation oncology should be represented in the tumor board. Site-specific surgical expertise should be obtained on a case-by-case basis for sarcomas arising at certain anatomical locations. |  | 7 |  |
| 5.5. | If a soft tissue sarcoma of the extremities is detected, the primary approach should be limb preservation. |  | 8 | 5.58, 5.81 |
| 5.6. | Resection of primary soft tissue sarcoma of the extremities should be performed as a wide resection. The goal is an R0 resection. | A | 13 | 5.29, 5.34, 5.41, 5.51 |
| 5.7. | The wound should be closed without tension. |  | 4 | 5.54, 5.8. |
| 5.9. | If a sarcoma grows to bony structures or is suspected, the periosteum of the affected bone section should also be resected. |  | 3 |  |
| 5.10. | In cases of urgent suspicion of infiltration of bone structures by a sarcoma, co-resection of the affected bone section should be generously indicated. |  | 4 |  |
| 5.12. | If there is evidence of blood vessel infiltration by sarcoma, the affected vessels should be segmentally resected and reconstructed. |  | 4 |  |
| 5.15. | Following resection of a primary sarcoma and histologic findings of R1 resection (other than ALT), presentation to a sarcoma center for consideration of resection should be made. |  | 8 | 5.26 |
| 5.16. | Patients with stage III soft tissue sarcoma should be offered multimodality therapy preoperatively as part of an multidisciplinary sarcoma board. |  | 5 |  |
| 5.20. | In the absence of evidence of lymphogenic metastasis, lymphadenectomy should not be performed as part of the primary tumor resection. |  | 7 | 5.23, 5.27 |
| 5.24. | Depending on resectability and histology, radical lymphadenectomy should be considered as resection therapy in cases of locoregional lymph node involvement of a corresponding sarcoma subtype. |  | 4 |  |
| 5.28. | Plastic reconstructive, especially microvascular, surgery is an essential therapeutic pillar for maintaining or restoring quality of life in soft tissue sarcomas of the head and neck. |  | 2 |  |
| 5.30. | Depending on the entity, tumor grading, and local recurrence rate, specific safety margins should be observed for cutaneous sarcomas. |  | 3 |  |
| 5.35. | Surgical treatment of retroperitoneal sarcoma should be performed in a sarcoma center with special expertise in surgery of RPS. |  | 2 |  |
| 5.38. | If R1 resection of soft tissue sarcoma of the retroperitoneum is found, re-resection should not be performed. |  | 3 |  |
| 5.44. | In leiomyosarcoma confined to the uterus, complete removal of the uterus should be performed without morcellement or uterine injury. |  | 4 | 5.48 |
| 5.59. | The planning of resection and plastic reconstruction should be made as an interdisciplinary therapeutic decision. |  | 3 |  |
| 5.85. | For soft tissue sarcomas with high risk of recurrence (> 5cm. deep-seated. G2/3) and chemosensitive subtype, neoadjuvant chemotherapy may be offered regardless of location. | O | 2 | 5.87, 5.88 |
